# Latent Profiles of Teacher-Reported Self-Regulation and Assessed Executive Function in Low-Income Community Preschools: Relations With Motor, Social, and School Readiness Outcomes

**DOI:** 10.3389/fpsyg.2021.708514

**Published:** 2021-09-27

**Authors:** Kate E. Williams, Laura A. Bentley

**Affiliations:** School of Early Childhood and Inclusive Education, Centre for Child and Family Studies, Queensland University of Technology, Brisbane, QLD, Australia

**Keywords:** self-regulation, executive function, early childhood, disadvantage, latent profile analysis, school readiness, preschool, behavior

## Abstract

This study contributes to understandings of early childhood self-regulation and executive function, and their components, through taking a person-centered approach to investigating how these skills cluster together in children aged 4–5years. A sample of children (*N*=206) from preschools in low socioeconomic communities were assessed through teacher report of self-regulation and three executive function tasks at the commencement of the preschool year. Outcome variables included teacher report of social skills and behavioral problems, and children’s school readiness and visual motor integration skills were directly assessed. When the scores from this low-income sample were compared to available norms, over 70% of children scored below the 50th percentile in executive function measures, approximately 20% were below average in self-regulation skills, 48% were delayed in school readiness scores, 36% had above average levels of internalizing problems, and 25% were above average in externalizing problems. A series of four latent profile models each used different measurement approaches and combinations of self-regulation and executive function components. In three of the four models (two which combined self-regulation and executive function measures and one with teacher report of self-regulation only), a high skill and low skill profile were found with 31 to 42% of children in the low profile depending on the model. Children were very similarly classified across all three models. When three executive function scores were modeled alone, a more complex three-profile solution emerged (low, moderate, and high) with 52% in the low profile. Children identified in the low profiles across all models were at greater risk of poorer school readiness, visual motor integration and social skills, and increased behavioral problems. Taken together, the findings suggest that self-regulation and executive function skills tend to cluster together at this age and in this low-income sample. Composite scores of teacher report of self-regulation are somewhat sufficient in identifying children who also have poorer executive function skills and are at risk of poorer motor, social, and school readiness outcomes. These children are an important target group for additional supports prior to school entry.

## Introduction

Self-regulation (SR) as an umbrella term is considered to include a wide range of processes that allow for the control of attention, cognition, emotion, and behavior in ways that are adaptive to circumstances and support goal attainment (Blair, 2016). The executive functions (EF) are specific cognitive control processes that in early childhood include dimensions of working memory (holding information in mind), shifting (flexible shifting of attention between information or tasks), and inhibition (the ability to control urges and resist distraction; Blair, 2016). Though stemming originally from different research domains (SR from the study of temperament and in particular effortful control, and EF from cognitive neuroscience), several recent models have sought to bring these constructs together to create a more complete understanding of self-regulatory development in early childhood (Blair, 2016; Bailey and Jones, 2019). Further, various bodies of research have sought to understand the extent to which these components are uni- or multi-dimensional in early childhood (Kälin and Roebers, 2021), how they are best measured (Camerota et al., 2020), and their shared and unique developmental course. Importantly, consistent evidence points to the role of early childhood SR (Robson et al., 2020) and EF (Finders et al., 2021) in supporting a range of positive life outcomes in achievement, social, and wellbeing domains. Further, for children living in socioeconomic disadvantage, it is understood that: SR and EF development are adversely impacted through the experience of early stressors and impacts of stress response physiology (Wesarg et al., 2020; Vogel et al., 2021); poorer skills in these areas are likely the key mechanism through which early poverty yields school readiness and ongoing achievement gaps (Perry et al., 2018a); and that stronger SR skills may offer some buffering of the effects of early risk (Crespo et al., 2019; Beisly et al., 2020).

In this study, we contribute to the above body of work through exploring profiles of SR and EF using various combinations of measures, in a sample of low-income preschool children. To date, most research on early childhood SR and EF has been variable-centered in nature. That is, correlation or regression analyses which assume that associations found hold for each individual within the research population. In contrast, person-centered approaches hypothesize that the population is heterogeneous in respect to the relationships between variables, and the way they cluster together in individuals (Collins and Lanza, 2009). In this study, we use person-centered latent profile analysis of various teacher-report measures of SR and assessed EF for a group of preschool children in low socioeconomic communities. This provides an opportunity to explore whether qualitatively different profiles can be identified in preschool children using these measures and how profile membership is associated with a range of other motor, social, and school readiness outcomes. In doing so, the study offers insights into how SR and EF skills cluster together in this sample, and how different combinations and forms of measures have utility in identifying children with similar skill profiles, and those at risk of poorer outcomes.

### Integrative Models of Self-Regulation and Executive Function, and Their Role in Development

The relation among SR and EF has been described as bidirectional. Specifically, emotional and attentional regulation, along with stress response physiology, are considered bottom-up self-regulatory processes characterized by their automatic and stimulus-driven nature (Nigg, 2017). Conversely, executive functioning is described as top-down, as it involves conscious cognitive processing to address novel problems or respond to internal mental representations, such a goals or rules (Ursache et al., 2012). When bottom-up aspects are functioning in an optimal range, executive functioning is facilitated, which in turn enhances attentional and emotional regulation, creating a bidirectional feedback loop (Blair, 2016). Executive attention is seen as an integral component within the system that can be both automatic and volitional, and somewhat provides a bridge between bottom-up processes, and the enactment of volitional higher-order processes (Blair, 2016). Building on this bidirectional model, a SR “gestalt” has been proposed (Bailey and Jones, 2019), which suggests that inhibition, shifting, and working memory (EFs), along with attentional control are core processes that facilitate cognitive, emotional, and social regulatory domains, which in turn support learning. In this way, teacher-report measures of childrens’ observed self-regulatory behavior in classroom settings (often considered in domains of cognitive, emotional, and social) reflect the behavioral enactment of underlying EF processes. However, Blair (2016) cautions that EF, while important for SR, is not synonymous with it. Specifically, patterns of association between bottom-up and top-down aspects of SR may differ across individuals. That is, behavior as observed and reported by teachers may be volitionally controlled by some children (employing EFs), while more automatically regulated by others through use of bottom-up attentional and emotional regulation strategies (Blair, 2016).

Independent of each other, various components of SR and EF have been linked with a range of positive life outcomes as well as risks. When considered together in the same model, adult-reported SR skills and assessed EF tend to each uniquely and in combination show predictive utility in terms of measures of school readiness (Vitiello and Greenfield, 2017) and achievement (Blair et al., 2015; Finders et al., 2021). Further, when EF components have been modeled separately (rather than as a composite), some differentiated findings have been reported. For example, off-task behavior as observed in the classroom has been linked with specific EFs (Moffett and Morrison, 2020). Children with lower inhibition scores were more likely to show off-task behavior through moving to other activities, while children with lower working memory scores and poorer attentional control tended to spend more time in disengaged off-task behavior. When components of EF are examined in relation to achievement, working memory tends to be the most highly predictive of achievement (Ahmed et al., 2019; Waters et al., 2021). Importantly, poorer SR and EF have been linked with greater risk of internalizing and externalizing behavior problems (Sulik et al., 2015; Perry et al., 2018b; Ip et al., 2019) and are also considered to co-develop with other important developmental areas including motor skills (Liew et al., 2018; McClelland and Cameron, 2019), which all impact on children’s learning trajectories.

### Self-Regulation, Executive Function, and Socioeconomic Status

It is well established that socioeconomic status and children’s SR and EF skills are correlated, indeed poorer SR and EF are considered key mechanisms through which socioeconomic gradients in school entry competencies and ongoing learning trajectories arise (Perry et al., 2018a). Stress response physiology is considered particularly integral to explaining these phenomena. Living in socioeconomic disadvantage can elevate children’s stress hormones which can impair neural regions associated with SR (Arnsten et al., 2015; Crespo et al., 2019). Together, these processes impair children’s SR capacity through progressively altering their emotional (Gustafsson et al., 2013; Raver et al., 2013) and cognitive and behavioral responses (Blair and Raver, 2012; Blair and Raver, 2016).

Importantly, not all children are affected equally by socioeconomic disadvantage and high levels of SR can act as a protective factor. For instance, SR has been shown to moderate the relationships between socioeconomic disadvantage and behavioral problems, where only children with lower SR display more behavioral problems (Kim-Spoon et al., 2012; Crespo et al., 2019). As the first 5years of life are a critical period of SR development (Montroy et al., 2016), this highlights the importance of identifying SR challenges at preschool entry in order to provide children with adequate support to improve these skills which may offer some protection against poorer outcomes.

### Measuring Early Childhood Self-Regulation and Executive Function

A range of research has examined measurement approaches to SR and EF and has sought to understand whether the constructs are uni- or multi-dimensional and which measurement methods and modeling approaches are most appropriate for particular purposes. Both constructs have been measured through adult report, observation, and direct assessment, each with affordances and limitations (McCoy, 2019) meaning there is currently no “gold standard” measure for either construct. Many researchers address this through the use of multiple measures and measurement approaches within the one study, which affords the opportunity to factor analyze components. In relation to the EFs, it is increasingly argued that in early childhood only a single construct is identifiable (Hartung et al., 2020) and that the most appropriate measurement approach is not latent variable modeling but instead composite scores (Willoughby and Blair, 2016; Camerota et al., 2020). SR measures are often conceptualized along the lines of Bailey and Jones’ (2019) model with factors for emotional, attentional/cognitive, and behavioral regulation (Howard and Melhuish, 2016; Howard et al., 2019).

When measures of SR and EF are factor analyzed together, some studies with children aged under 7years find a single factor solution (Dilworth-Bart et al., 2018; Kälin and Roebers, 2021) while others find separable SR and EF factors (Neuenschwander et al., 2012). Many studies that include measures of adult-reported and assessed SR/EF do not undertake factor analysis of the measures in combination but rather develop separate scores for each construct and each measurement approach. This is likely due to conceptual separation of the constructs by researchers and/or recognition that performance tasks and rating scales are likely to measure different aspects or levels of the same construct, under very different conditions (Toplak et al., 2013). Cross-sectionally, when assessed EFs are correlated with adult-reported SR, observational measures of task persistence or assessments of effortful control (related to SR) estimates are typically moderate (0.2 to 0.4; Blair et al., 2015; Vitiello and Greenfield, 2017; Oeri et al., 2020). This suggests that there are important distinctions across constructs and/or that the differences created by various measurement approaches are substantial.

### Person-Centered Approaches to Understanding Early Childhood Self-Regulation and Executive Function

In this section, we review the handful of recent studies that have included constructs related to SR and EF in a person-centered approach as we do in the current study. These approaches have the potential to offer unique insights into how these constructs cluster together in young children. Inferences can also be made about which measures and in what form are able to distinguish the most at-risk profiles, with this identification important if early intervention is to be appropriately targeted.

To our knowledge, only one prior study has used latent profile analysis with a combination of measures conceptualized as SR and EF, in low income, typically developing preschool children as investigated in the current study (Bayly and Bierman, 2021). Data on four EF tasks (conceptualized to represent cognitive regulation), and a teacher report of SR from a school readiness survey (conceptualized as behavioral regulation, with two subscale scores used), were profiled for 566 Head Start children in the pre-kindergarten year (Bayly and Bierman, 2021). Four profiles were found with 30% of children displaying pervasive dysregulation across cognitive and behavioral regulation. A further 22% showed behavioral dysregulation without cognitive dysregulation, 29% had average regulation across both constructs, and 18% were in the high regulation profile. Taken together in this low-income sample, a total of 52% of children were classified in dysregulated profiles; however, two distinct clusters of dysregulation were found. Over half of the children were dysregulated according to both EF assessments and SR adult reports, and the remaining showed average tested EF skills, but poor behavioral dysregulation as per teacher report. Importantly, both dysregulation groups showed reading achievement, teacher-rated academic performance, and attentional problems that were similar across dysregulation groups, but significantly poorer compared to the regulated groups, with gaps continuing to Grade 2. This suggests that even when assessed EF skills are average (as they were in one of the dysregulated profiles), if these are not combined with self-regulatory skills as observed in the classroom by teachers, then learning is likely to be impacted.

Another profiling study with kindergarten children (*N*=15,770) included additional profile indicators of reading and math achievement, along with EF (single shifting task) and SR (single score on teacher report). Findings also suggested that behavioral aspects of SR are important for learning (Elliott, 2019). Again, a four-profile solution was reported with a total of 51% of children classified in a profile with average scores across all measures, and 11% in a profile with average EF and SR and high achievement scores. The remaining 38% were in profiles with various combinations of SR and EF skills with 23% in a profile with low achievement, average EF, and poor SR and 15% in a profile with low achievement, low EF, and moderately low SR. When linking to Grade 3 academic outcomes, the kindergarten profile that included poor SR was more problematic than the profile characterized by poor EF, reinforcing the important role of classroom SR as observed and reported by teachers in predicting ongoing learning trajectories.

Another study with a general population sample of kindergarten children aimed to investigate patterns of EF performance of 10,700 children in the United States (Litkowski et al., 2020) but also included measures that have been conceptualized as SR in other studies. Data analyzed included assessed working memory and attention shifting, and teacher-reported inhibition, attentional regulation, and approaches to learning (representing regulated behavior in a classroom learning environment). A five-profile solution was found with 38% of children in a high EF profile, 33% in an average profile, 7% in a profile showing low scores on direct assessments but high scores on teacher report, 15% in a profile with mixed assessment scores and low teacher ratings, and 7% in a profile with poor skills across measures and labeled as vulnerable. When correlated with Year 3 achievement, children in the high EF profile had the highest math and reading scores, and children in the vulnerable profile had the lowest. An interesting finding was that children in the fourth profile labeled “mixed direct assessments – low teacher ratings” demonstrated significantly lower math and reading scores in Year 3, compared to children in the third profile, “low direct assessment – high teacher ratings.” This is again congruent with the findings of the prior two studies described here suggesting high EF alone may not ensure learning achievement unless children also demonstrate strong enacted classroom SR.

A range of other latent profile studies with young children that have included SR and/or EF measures, often along with other developmental constructs including motor, language, and social skills have typically yielded solutions of two to four profiles (Denham et al., 2012; Kia-Keating et al., 2018; Usai et al., 2018; Houwen et al., 2019; Jacob et al., 2019; Sparapani et al., 2019). Across person-centered analyses in early childhood, where covariates are explored, boys (Kia-Keating et al., 2018; Teivaanmäki et al., 2019), children from lower socioeconomic homes (Denham et al., 2012; Bayly and Bierman, 2021), children with poor visual motor coordination (Houwen et al., 2019), children with clinical diagnoses including Autism Spectrum Disorder (ASD) and Attention Deficit Hyperactivity Disorder (ADHD Ros and Graziano, 2019; Baez et al., 2020), and children who display internalizing and externalizing behaviors (Kia-Keating et al., 2018) are more likely to be classified within lower-performing SR/EF profiles. Further, profile membership tends to be associated as expected with a range of outcomes. Specifically, children in profiles characterized by a constellation of poorer cognitive, motor, and social skills are more likely to experience poorer longitudinal outcomes in a range of areas including overall academic achievement (Denham et al., 2012) and mathematic performance (Usai et al., 2018).

### The Current Study

The current study is a cross-sectional observational study in which we use a person-centered approach (latent profile analysis) to understand the clustering of teacher-reported SR and assessed EF skills in a sample of low-income children at the beginning of their preschool year, and associations between profile membership and a range of motor, social, and school readiness measures. To our knowledge, this is only the second study to do this, and importantly, the first latent profile study to run multiple analyses using different approaches to representing SR and EF, including scores for their sub-components, and total composite scores. While prior latent profile analysis (LPA) studies described above variously included direct measures of EF, and teacher-reported SR as we do, this study is distinguished by a thorough, yet exploratory, approach in which three measures of EF (shifting, working memory, and inhibition) that align with current understandings of the nature of EF in early childhood, along with a comprehensive teacher report on self-regulation is subjected to a range of person-centered analyses. Bayly and Bierman (2021) used the components of EF and SR as discrete subscales (Bayly and Bierman, 2021) despite recent recommendations that composite scores may be the most accurate representation of each EF and SR (Willoughby and Blair, 2016; Camerota et al., 2020). By taking this exploratory approach, this study sheds light on: (1) the prevalence of SR and EF skill problems in low-income Australian children; (2) the utility of various combinations and forms of SR and EF indices to identify groups of children showing similar constellations of behavior which may be associated with outcomes in a range of domains.

The research questions are:

1. What are the profiles of SR and EF discoverable using four different sets of indicators as follows:
  - Model 1: composite scores for SR (yielded from three teacher-reported subscales) and EF (yielded from three direct assessments).
  - Model 2: component items which include a total of three SR subscales and three EF measures.
  - Model 3: three teacher-reported SR subscales only.
  - Model 4: three directly assessed EF measures only.
2. How is profile membership associated with a range of child skills including school readiness, visual motor integration, and social emotional behavioral development?
3. To what extent do each of the modeling approaches similarly classify children into risky profiles?

## Materials and Methods

This study uses the baseline data only from a larger RCT study (Williams et al., 2020) focused on the effectiveness of an intervention for children’s SR development. Ethical clearance was gained through a University Human Research Ethics Committee, Queensland University of Technology Human Research Ethics Committee, approval 1900000566, and the trial is registered with the Australian New Zealand Clinical Trials Registry, ACTRN12619001342101.

### Participants

Eight early childhood centers, which enrolled preschool-aged children (4–5years), participated. Centers were invited based on: a community score in the bottom three deciles of a national index based on census data that positions areas in Australia according to relative socioeconomic advantage and disadvantage (Australian Bureau of Statistics, 2018); located in a community area where 2018 Australian Early Development Census data indicated a higher than national average level of child vulnerability in both domains of social competence and emotional maturity (Australian Government Department of Education and Training, 2019). In February 2020, at the commencement of the preschool year, teachers invited all enrolled families to consent to research participation. Across centers, of the potential 228 child participants, parental written consent was gained for 211 children (96%). Of these, five children left the preschool prior to data collection commencing or were absent during the data collection phase leaving a final analytic sample of 206.

Children ranged in age from 44 to 67months with a mean age of 50.56months (*SD*=4.47), 51% (*n*=104) were female, 17% identified as Aboriginal (*n*=34), and 16% spoke a language other than English at home (*n*=32; 24 different languages represented) with the languages spoken by two or more children being Arabic, Karen, Urdu, Kurdish, Punjabi, Tongan, Turkish, Vietnamese, and Zomi. Caregivers ranged in age from 17 to 67 with a mean age of 33years (*SD*=6.6). A total of 30% were single (*n*=57), 26% (*n*=48) had not completed high school, 25% (*n*=47) had a university degree, and 60% of households earned $1,000AUD a week or less. There were 13% of parents (*n*=26) who identified concerns with their child’s development with most related to suspected or diagnosed speech delay, ASD, or ADHD.

### Procedure

Parents completed socio-demographic surveys at recruitment in February 2020, and across 2weeks in March 2020, validated baseline SR (teacher report) and EF measures (direct assessments with children) were collected. Assessors visited preschools in pairs and withdrew children to undertake assessments, with each child working with each assessor for a 15-min period (total of 30min of assessments per child split across two sessions with a break in between).

### Measures

Executive function. Three EF measures from the *Early Years Toolbox* (EYT) iPad tasks were used (Howard and Melhuish, 2016). These tasks have shown good convergent validity, correlating with other established measures tapping the same constructs with full psychometric details documented previously (Howard and Melhuish, 2016). Scores on each of the three assessments were standardized and used as individual scores in some models, and also summed and averaged to create a component EF score for other analyses (*α*=0.56). We note this alpha estimate as low but not unexpected given recent arguments that the nature of EF as a construct is that multiple measures used together represent formative, rather than reflective, indicators of the latent construct of EF. Thus, EF is not best understood or modeled in ways that are concerned with shared variance across measures (e.g., latent variable models) but total variance (e.g., sum scores as used here; Camerota et al., 2020).

Working memory was measured through the EYT *Mr. Ant task*, which measures visual-spatial working memory. Children were asked to remember the spatial locations of “stickers” placed on a cartoon ant and identify these locations after a brief retention interval. Test trials increased in difficulty as the task progressed, with three trials at each level of complexity (progressing from one to eight stickers). The possible score range was 0–8.

Inhibition was measured using the EYT *Go/No-Go task*, which required participants to tap the screen on “go” trials (“catch the fish”) and not tap the screen on “no-go” trials (“avoid catching sharks”). As most stimuli were “go” trials (80% fish), this generated a prepotent tendency to respond, requiring participants to inhibit this response on no-go trials (20% sharks). Inhibition was indexed by an impulse control score that is the product of proportional “go” (to account for the strength of the prepotent response generated) and “no-go” accuracy (to index a participant’s ability to overcome this prepotent response) with a possible range of 0–1.

Shifting was measured using the EYT *Card Sorting task* based on the protocols of the commonly used Dimensional Change Card Sort task (Zelazo, 2006). Children were required to sort cards (i.e., red rabbits and blue boats) by a sorting dimension (i.e., color or shape) into one of two locations (identified by a blue rabbit or a red boat), and then switch to the alternate sorting rule. Scores represented the number of correct sorts after the switch phase with a possible range of 0–12.

Self-regulation was measured through teacher report on three subscales of the EYT *Child Self-Regulation and Behavior Questionnaire* (CSBQ). The CSBQ is a 33-item educator-report (or parent report) questionnaire that yields seven subscales. Each item requires the respondent to evaluate the general frequency of target behaviors, on a scale from 1 (*not true*) to 5 (*certainly true*). Three subscales were used to represent self-regulation. The Cognitive Self-Regulation subscale has five items (*α*=0.87) including “persists with difficult tasks” and reflects persistence or attentional regulation. The Behavioral Self-Regulation subscale has five items (*α*=0.91) including “waits their turn in activities.” The Emotional Self-Regulation subscale has six items (*α*=0.83) including “gets over being upset quickly.” These scores were used in two forms across models: (1) for each subscale, scores were summed and averaged, and these scores were standardized; (2) to create a composite SR score, these standardized scores for each subscale were then summed and averaged (*α*=0.87).

School readiness was measured by the Bracken School Readiness Assessment—Third Edition which evaluates concepts essential to early communication development and school readiness (Bracken, 2007). The School Readiness Composite comprises six subtests (colors, letters, numbers/counting, sizes, comparisons, and shapes) and assesses children’s knowledge of concepts traditionally taught to children in preparation for formal education. Raw total correct scores were used as an outcome variable with a potential score range of 0–160.

Visual motor integration was measured using the Design Copy task from the *Early Screening Inventory* (ESI-R; Harrison, 1990). The visual motor task from the ESI requires children to use a pencil to copy geometric shapes pictured on cards, such as a square or circle. The Design Copy task was double scored (blind) by the lead author and a single research assistant, with all discrepancies in scores discussed in line with scoring protocol and resolved for a final agreed score. Each of the six tasks is scored on a scale of zero to 2 with a possible total score range of 0–12.

Other social emotional outcomes included were also measured with the teacher-reported EYT CSBQ (Howard and Melhuish, 2016) and included the total subscale scores for: Internalizing Problems (e.g., “most days distressed or anxious”; 5 items, *α*=0.78); Externalizing Problems (e.g., “aggressive to children”; 5 items, *α*=0.84); Prosocial Behavior (e.g., “helps others,” 5 items, *α*=0.91); and Sociability (e.g., “chosen as a friend by others,” 7 items, *α*=0.90).

Socio-demographic variables were derived from parent surveys and included child gender (0=male; 1=female), child age in months, Aboriginal status (0=no; 1=yes), non-English home language (0=no; 1=yes), developmental delay (0=no; 1=yes), parental complete high school education (0=incomplete; 1=completed high school), and low income (0=> $1,000AUD per week; 1=< $1,000 per week).

### Approach to Analysis

These analyses take a person-centered analytic approach. First, we used LPA in MPlus Version 8.2 (Muthén and Muthén, 1998–2017) to establish profiles of SR and EF using the same sample but different profile indicators. LPA is a semi-parametric group-based approach that allows for estimation of qualitatively different groups when membership cannot be observed *a priori* (Ferguson et al., 2020). In Model 1, we used composite scores for SR (yielded from three teacher-reported subscales) and EF (yielded from three direct assessments). In Model 2, we use the component items which included a total of three SR subscales and three EF measures. In Model 3, we used the three teacher-reported SR subscales only, and in Model 4, we use the three directly assessed EF measures only.

Selection of the optimal number of profiles for each cohort was based on three measures of relative model fit (compared to the same model with one less profile): The Bayesian Information Criterion (BIC); Consistent Akaike’s Information Criterion; and the Lo-Mendell-Rubin (LMR) test. In addition, we examined the classification probabilities of children into profiles, and in particular considered solutions with respect to the smallest profile classification given that profile solutions in which very small numbers of children are likely classified are unlikely to be replicated in other samples or in real-world contexts (Ferguson et al., 2020).

As a second step, we exported class membership probabilities to the Statistical Package for Social Sciences program Version 27 (IBM Corp, 2020), assigning to each child the profile for which they had the highest probability. Differences across profiles were then examined in relation to socio-demographic variables and a range of other variables including visual motor integration and school readiness assessment scores and teacher-reported social skills and behavioral problems (ANOVAs for continuous variables and chi-square tests for binary variables).

Missing data were negligible for the measures used as profile indicators with the highest level of missing data being 6.8% for inhibition. For socio-demographic variables considered in relation to profile membership, there was a maximum of 18% missing data on household income, where parents chose not to provide this information on their socio-demographic survey. For outcome variables, there was a maximum of 4% missing. For the latent profile analysis, we used the MLR estimator in MPlus which provides full information maximum likelihood estimates with robust standard errors to account for missing data.

## Results

### Preliminary Analysis

Exploratory factor analysis including three SR subscale scores and all three measures of EF showed a two-factor model was the best fit for the data with separable factors for SR and EF. Therefore, scores for each EF measure were standardized, summed, and averaged to create an overall EF score in line with recent recommendations regarding composite approaches (Camerota et al., 2020).

### Descriptive Statistics

Bivariate correlations and descriptive statistics for all variables are shown in Supplementary Table S1. On average girls had better shifting, SR, and social skills. Older children had better EF, visuo-motor skills, school readiness, SR, and social skills. Having a parent with a complete high school education was correlated with more household income, and higher cognitive SR, and school readiness. Low family income was correlated with lower shifting skills, school readiness, and prosocial skills. Aboriginal children had lower household income and lower school readiness scores and children from a non-English home language had significantly poorer EF, school readiness, and social skills. Children with developmental delay had poorer inhibition, SR, social skills, and more internalizing and externalizing behaviors. Self-regulation subscales were highly correlated with each other, while EF task scores were moderately correlated with each other and with SR measures. Both SR and EF were correlated with outcome measures in the expected direction.

Next, we used published norms on the measures used in the study, where available, to understand the relative skills of this sample of children living in low-income areas compared to what might be expected from a representative sample. As seen in Table 1, while 78 to 85% of children in the sample scored close to the Australian average on teacher-reported measures of SR (Howard and Melhuish, 2016), most children in this sample scored poorly for their age in the EF tasks. Specifically, almost 87% of children scored below the 25th percentile of Australian norms in working memory, 71% in inhibition, and most children (93%) scored in the 25th to 50th percentile range for shifting for their age. In relation to the outcome measures, a total of 48% of children were delayed or very delayed on school readiness (Bracken, 2007), 24% were below average in sociability and prosocial skills, 16% had elevated externalizing problems, and 26% had elevated internalizing problems.

**Table 1:** Percentage of sample in each of normed percentile groups for EF, SR, and outcome measures where norms available.

	Normed percentile group			
	Very low, 5th percentile	Low, 10th percentile	Slightly low, 20th percentile	Close to average
Behavioral SR	8.8	4.9	8.3	77.9
Cognitive SR	5.4	4.4	11.3	78.9
Emotional SR	5.4	4.9	4.9	84.8
	**Below 25th percentile**	**25th to 50th percentile**	**50th to 75th percentile**	**Above 75th percentile**
Working memory	86.8	0	3	10.2
Inhibition	71.4	12.5	10.9	5.2
Shifting	0.5	92.9	5.6	1
	**Very delayed**	**Delayed**	**Average**	**Advanced**
School readiness	12.2	35.7	46.4	5.6
	**Very low**	**Low**	**Slightly low**	**Average**
Sociability	10.3	2	13	74.5
Prosocial	4.9	7.4	11.3	76.5
	**Very high**	**High**	**Slightly high**	**Close to average**
Externalizing problems	5.4	3.9	17.2	73.5
Internalizing problems	8.3	7.8	19.6	64.2

SR, self-regulation.

### Profile Selection and Description

**Figure 1:**
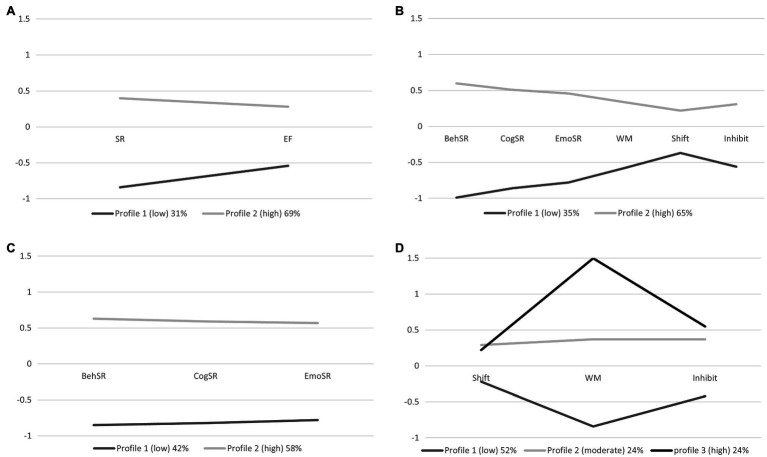
Average indicator scores for profiles across four latent profile analysis models. **(A)** Model 1 of composite scores SR (yielded from three teacher-reported subscales) and EF (yielded from three direct assessments). **(B)** Model 2 of component items which include a total of three SR subscales and three EF measures. **(C)** Model 3 of three teacher-reported SR subscales only. **(D)** Model 4 of three directly assessed EF measures only. SR, self-regulation composite; EF, executive function composite; Beh SR, behavioral self-regulation; Cog SR, cognitive self-regulation; Emot SR, emotional self-regulation; WM, working memory; Shift, shifting; and Inhibit, inhibition.

Table 2 presents the latent profile results for the four modeling approaches taken. **For Model 1 (SR and EF composite scores as indicators),** a **two-profile** solution was selected as the best fit given it had the lowest BIC value, and the non-significant LMR test for the three-profile solution suggested that an additional profile did not improve model fit. Classification probabilities show that 92% of children in Profile 2 were correctly classified, while only 78% of children in Profile 1 were correctly classified. Profile 1, which we have called **“low” (31% of the sample)**, was characterized by below mean scores on both SR and EF composites, and Profile 2, which we call **“high” (69% of the sample)**, was characterized by above mean scores on both SR and EF (see Figure 1A). There was significant separation of profiles on both the composite SR and EF scores used as indicators for the profile analysis, and the component subscales and assessments (See Table 3).

**Table 2:** Model fit information for the latent profile analyses.

	Number of profiles	Log-likelihood (LL)	Number of parameters	BIC	AIC	LMR *value of p*	N (%) classified into smallest profile
Model 1: Composite scores for SR and EF	1	−473.04	4	967.39	954.08		
	**2**	**−456.59**	**7**	**950.48**	**927.19**	**0.01**	**63 (30.6)**
	3	−449.73	10	952.73	919.45	0.65	35 (18.1)
	4	−444.15	13	957.57	916.38	0.06	11 (5.3)
Model 2: Component scores (3 scores for SR; 3 scores for EF)	1	−1699.21	12	3462.42	3422.42		
	**2**	**−1567.43**	**19**	**3286.09**	**3172.86**	**0.00**	**73 (35.4)**
	3	−1516.01	26	3170.55	3084.03	0.10	41 (19.9)
	4	LL not replicated					
Model 3: Teacher-report self-regulation components only (3 scores)	1	−866.79	6	1765.48	1745.57		
	**2**	**−767.05**	**10**	**1587.29**	**1554.11**	**0.02**	**85 (41.7)**
	3	−720	14	1514.69	1468.24	0.30	31 (15.2)
	4	−693.30	18	1482.32	1422.59	0.00	11 (5.3%)
Model 4: EF components only (3 scores)	1	−832.43	6	1696.61	1676.85		
	2	−774.11	10	1601.16	1568.23	0.00	82 (41.2)
	**3**	**−723.25**	**14**	**1520.60**	**1474.49**	**0.00**	**47 (23.6)**
	4	−686.52	18	1468.32	1409.04	0.00	13 (6.5)
	5	−672.74	22	1461.94	1389.48	0.01	13 (6.5)
		LL not replicated					

**Table 3:** Profile differences on indicator measures and age norm groups.

	EF	SR	Beh SR	Cog SR	Emot SR	WM	Inhibition	Shifting
	*M (SD)*		*M (SD)*/% in average norm group or above 50th percentile for EFs					
**Model 1: Composite SR and EF scores**								
Profile 1 (low) *n*=64 (31%)	−0.66 (0.42)	−0.98 (0.62)	−1.08 (0.67)/37%	−0.93 (0.73)/51.6%	−0.92 (0.94)/61.3%	−0.74 (0.44)/1.7%	−0.71 (0.82)/1.8%	−0.53 (0.81)/0%
Profile 2 (high) *n*=142 (69%)	0.28^*^ (0.65)	0.44^*^ (0.60)	0.48^*^ (0.70)/95.8%^*^	0.41^*^ (0.81)/90.8%^*^	0.41^*^ (0.72)/95.1%^*^	0.31^*^ (0.09)/13.67%^*^	0.29^*^ (0.92)/6.6%	0.23^*^ (1.0)/1.4%
**Model 2: Component SR and EF**								
Profile 1 (low) *n*=72 (35%)	−0.54 (0.51)	−0.92 (0.60)	−1.05 (0.65)/ 37.5%	−0.89 (0.73)/51.4%	−0.81 (0.98)/63.8%	−0.63 (0.63)/2.9%	−0.61 (0.85)/ 1.5%	−0.41 (0.86)/0%
Profile 2 (high) *n*=134 (65%)	0.29^*^ (0.67)	0.51^*^ (0.55)	0.59^*^ (0.60)/100%^*^	0.49^*^ (0.77)/93.9%^*^	0.45^*^ (0.69)/96.2%^*^	0.34^*^ (1.0)/14%^*^	0.31^*^ (0.92)/7.1%	0.22^*^ (1.0)/1.6%
**Model 3: Teacher-report SR only**								
Profile 1 (low) *n*=87 (42%)	−0.28 (0.67)	−0.84 (0.58)	−0.88 (0.73)/47%	−0.83 (0.72)/ 54%	−0.81 (0.90)/64.7%	−0.37 (0.86)/5%	−0.31 (1.04)/6.5%	−0.23 (0.86)/0%
Profile 2 (high) *n*=119 (58%)	0.21^*^ (0.71)	0.61^*^ (0.61)	0.64^*^ (0.60)/100%^*^	0.60^*^ (0.71)/96.6%^*^	0.59^*^ (0.56)/99.2%	0.26^*^ (1.01)/13.9%^*^	0.21^*^ (0.92)/4.4%	0.17^*^ (1.07)/1.7%
**Model 4: Tested EF**								
Profile 1 (low) *n*=107 (52%)	−0.48 (0.48)	−0.32 (0.89)	−0.43 (1.0)/60.6%	−0.30 (0.95)/73%	−0.22 (1.05)/80.8%	−0.84 (0.15)/0%	−0.42 (0.92)/2%	−0.22 (0.94)/0%
Profile 2 (moderate) *n*=49 (24%)	0.35^^^ (0.55)	0.44^#^ (0.70)	0.52^#^ (0.70)/97.9%	0.44^#^ (0.92)/89.4%	0.36^#^ (0.84)/93.6%	1.5^^^ (0.21)/0%	0.55^#^ (0.88)/22%	0.22^#^ (1.12)/12.7%
Profile 3 (high) *n*=49 (24%)	0.75^^^ (0.49)	0.35^#^ (0.70)	0.50^#^ (0.71)/97.8%	0.32^#^ (0.93)/84.8%	0.22^#^ (0.84)/89.1%	0.37^^^ (0.38)/42.6%	0.37^#^ (0.89)/30%	0.29^#^ (0.91)/4.2%
Profile 2 and 3 combined *N*=98 (48%)	0.55^*^ (0.56)	0.40^*^ (0.70)	0.52^*^ (0.70)/97.85%^*^	0.38^*^ (0.92)/87%^*^	0.29^*^ (0.84)/91.4%^*^	0.93^*^ (0.65)/21.3%^*^	0.46^*^ (0.88)/ 8.8%^*^	0.25^*^ (1.01)/2.1%

EF, executive function composite; WM, working memory; SR, self-regulation composite; Beh SR, behavioral self-regulation; Cog SR, cognitive self-regulation; and Emot SR, emotional self-regulation;

* significantly different from the low profile;

# significantly different from the low profile but not different from each other;

^ significantly different from the low profile and all other profiles in the group.

For **Model 2 (component indicators of SR and EF),** a **two-profile** solution was also selected due to an elbow in the BIC values when plotted, and the non-significant LMR test for the three-profile solution suggested that an additional profile did not improve model fit. Classification probabilities showed that 92% of children in Profile 1 and 98% of children in Profile 2 were correctly classified. Similar to Model 1, Profile 1, which we call **“low” (35% of the sample)**, was characterized by below mean scores on all component measures of SR and EF. Profile 2, which we call **high (65% of the sample)**, was characterized by above mean scores on all component measures of SR and EF (see Figure 1B). There was significant separation of profiles, based on profile averages, on all SR and EF component and composite scores (see Table 3).

For **Model 3 (teacher report on SR subscales only),** a **two-profile** solution was also selected due to an elbow in the BIC values when plotted, and the non-significant LMR test for the three-profile solution suggested that an additional profile did not improve model fit. Classification probabilities showed that 92% of children in Profile 1 and 95% of children in Profile 2 were correctly classified. Again, like Models 1 and 2, Profile 1 (called **low with 42% of the sample**) had below mean scores on all teacher-reported components of SR, and Profile 2 (called **high, 58% of the sample**) had above mean scores on all teacher-reported components of SR (see Figure 1C). Across these profiles, there was significant separation of the profiles both on their average teacher-reported SR indices used as indicators in the profile analysis, and on the EF, measures not used in this model (see Table 3).

For **Model 4 (assessed EF components),** both the three and four-profile solution were considered. Although the BIC elbow was at the four-profile solution, the smallest profile in this solution was just 6.5% of the sample and so considered potentially not practicably meaningful. Further, inspection of classification probabilities across the three and four-profile solutions showed that the four-profile solution had largely replicated the three-profile solution but split one profile into two smaller groups. For these reasons, the more parsimonious **three-profile** solution was selected as the final solution. Classification probability showed that 100% in Profile 1, 98% of children in Profile 2, and 99% in Profile 3 were correctly classified. Profile 1 (we call **low**) **had 52% of the sample** and had below mean scores on all three EF indices. Profile 2 (**moderate, 24%**) and Profile 3 (**high; 24%**) had above mean scores on all three EF measures. There was less evidence of clear separation of profiles within this model. The most distinguishing indicator among the three profiles was working memory which showed a clear gradient across the low to high profiles. For inhibition and shifting, the moderate and high groups differed significantly from the low profile, but not from each other. In terms of the EF composite, there was significant gradient across all three groups with significant differences between the low, moderate, and high groups. For the SR composite, and each of the SR subscales, the low profile in this solution was distinguished from the other two profiles, which had similar scores to each other.

### Profile Comparisons: Socio-Demographic and Outcome Measures

Table 4 documents the socio-demographic differences among profiles. Across all profiles, girls were more likely to be in the higher skilled profiles, and children from non-English speaking homes and with developmental delay were more likely to be in the lowest profiles. For Models 2 to 4, children in the higher skilled profiles were on average slightly but significantly older than children in the low profiles. Aboriginal status was not associated with profile membership. There was a trend toward children in the higher skill profiles having parents with a higher education level and lower rates of extremely low income, but this was not statistically significant.

**Table 4:** Differences across profiles on socio demographics.

	Female %	ATSI %	NESB %	DD %	Age in months *M (SD)*	Caregiver finished high school %	Low income %
**Model 1: Composite scores for SR and EF**							
Profile 1 (low)	36.5	14.5	27.4	24.6	49.73 (4.73)	69.6	63
Profile 2 (high)	56.6^*^	18.2	10.6^*^	7.9^*^	50.92 (4.31)	76	59.1
**Model 2: Component scores (3 scores for SR; 3 scores for EF)**							
Profile 1 (low)	39.7	15.2	25	22.5	49.53 (4.71)	70	63.9
Profile 2 (high)	56.4^*^	18.11	10.6^*^	7.8^*^	51.12^*^ (4.24)	76	58.3
**Model 3: Teacher-report self-regulation components only (3 scores)**							
Profile 1 (low)	42.4	13.2	22.6	20.5	49.65 (4.82)	72	61.7
Profile 2 (high)	56^*^	19.3	11^*^	7.8^*^	51.16^*^ (4.13)	75	58.6
**Model 4: EF components only (3 scores)**							
Profile 1 (low)	41.9	16.5	23	17.6	49.71 (4.29)	71.3	65.1
Profile 2 (moderate)	66	14	4.3	8.7	51.06 (4.27)	78	45.9
Profile 3 (high)	51.1	17.4	6.5	8.9	52.19^*^ (4.65)	79	61.5
Profiles 2, 3, combined	58.5^*^	15.7	5.4^*^	8.7	51.63^*^ (4.47)	78.6	53.9

ATSI, Aboriginal and Torres Strait Islander; NESB, non-English speaking background; and DD, developmental delay. Low income=< $1000AUD/week;

* chi-square test comparing percentage across profiles or for child age F test of compared to low profile significant at *p*<0.05.

In relation to outcomes (Table 5), children in the higher skilled profiles across all models had significantly higher school readiness and visual motor integration skills, stronger prosocial and sociability scores, and fewer teacher-reporting externalizing and internalizing behavior problems. In comparing children across profiles in relation to available norm groups, across models, more children in the higher SR and EF skill profiles scored in the average or above average range on normed scores for school readiness and all social-emotional-behavioral indices.

**Table 5:** Differences across profiles on outcome measures.

	School readiness	VMI	Internalizing	Externalizing	Prosocial	Social
	*M (SD)*/% in average or above category compared to norms					
**Model 1: Composite SR and EF scores**						
Profile 1 (low)	23.53 (13.56)/30.5	1.22 (1.33)	2.58 (0.62)/35.5%	2.82 (0.89)/37.1	2.56 (0.78)/35.5	2.92 (0.91)/54.8
Profile 2 (high)	36.80^*^ (16.42)/61.3	2.49^*^ (1.94)	2.03^*^ (0.62)/76.8	1.56^*^ (0.64)/89.4	3.93^*^ (0.61)/94.4	3.67^*^ (0.80)/83.1
**Model 2: Component SR and EF**						
Profile 1 (low)	24.71 (13.65)/38.2	1.26 (1.31)	2.53 (0.62)/37.5	2.74 (0.88)/40.3	2.66 (0.79)/38.9	3.00 (0.91)/58.3
Profile 2 (high)	37.10^*^ (16.67)/59.4	2.57^*^ (1.96)	2.02 (0.62)/100	1.50^*^ (0.60)/100	4.0^*^ (0.66)/97	3.67^*^ (0.80)/83.3
**Model 3: teacher-report SR only**						
Profile 1 (low)	27.58 (15)/47.5	1.64 (1.61)	2.55 (0.67)/37.6	2.66 (0.88)/42.4	2.74 (0.77)/47.1	2.97 (0.87)/57.6
Profile 2 (high)	36.23^*^ (16.95)/55.3	2.49^*^ (1.95)	1.95^*^ (0.54)/83.2	1.43^*^ (0.55)/95.8	4.07^*^ (0.62)/97.5	3.77^*^ (0.77)/ 86.6
**Model 4: tested EF only**						
Profile 1 (low)	25.36 (14.12)/33.7	1.39 (1.51)	2.31 (0.61)/57.7	2.21 (0.98)/62.5	3.16 (0.95)/63.5	3.26 (0.86)/70.2
Profile 2 (moderate)	28.66^#^ (14.74) 68.1	2.77^#^ (1.81)	2.05 (0.65)/74.5	1.56^#^ (0.80) 85.1	4.0 (0.71)^#^ 3/ 93.6	3.81 (0.71)^*^/87.2
Profile 3 (high)	43.53^#^ (15.68)/76.1%	3.06^#^ (1.99)	2.04 (0.73) 71.7	1.68^#^ (0.63)/89.1	3.89 (0.76)^#^/91.3	3.57 (0.97)/73.9
Profiles 2, 3, combined	41.10^*^ (15.33)/72	2.91^*^ (1.90)	2.04^*^ (0.69)/73.1	1.62^*^ (0.72)/87.1	3.96^*^ (0.74)/92.5	3.69^*^ (0.85)/80.6

VMI, visual motor integration;

* *F* test of mean differences comparing score to low profile significant at *p*<0.05.

# Significant difference to the low profile but not to each other.

### Classification of Children Across Different Models

In this section, we compare classification of children into profiles across the different models with Table 6 showing cross-tabulation of profile allocation for each model. Comparing Model 1 (composite SR and EF scores) to Model 2 (component subscales indices for SR and EF) shows all children classified into the same profiles across the Models except for 10 children (4.9%). Specifically, 10 children who were classified in the high profile in Model 1 (composite scores) were classified in the low profile in Model 2 (component scores).

**Table 6:** Cross-tabulation of classification of children into profiles across models.

	Model 2 low profile	Model 2 high profile	Model 3 low profile	Model 3 high profile	Model 4 Low	Model 4 moderate/high
Model 1 low profile	63	0	59	26	55	5
Model 1 high profile	10	133	3	116	50	89
Model 2 low profile			69	3	61	9
Model 2 high profile			16	116	44	85
Model 3 low profile					60	22
Model 3 high profile					44	71

Low profile refers to the profile established in the latent profile analysis as having lowest SR and EF compared to the high profile. See Table 3 for exact groupings and estimates for these profiles.

Comparing Model 1 (composite SR and EF scores) to Model 3 (teacher-reported SR subscales) shows again that most children (86%) were classified into the same profiles across the models. Differences were that three children classified in the high profile through Model 1 were classified in the low profile by Model 2, and 26 children (12.7%) that were classified in the low profile in Model 1 were classified in the high profile in Model 2. Comparison of Model 2 and Model 3 shows highly similar findings, not surprising given Model 1 and Model 2 largely produced the same profile groupings for children.

Comparing Model 1 to 4 (EF scores) shows that classification of children in the low profile by Model 1 was largely replicated by Model 4 with only five children classified as low in Model 1 but in the moderate and high profiles in Model 4. However, 44% of cases who were classified into the “high” profile in Model 1 were classified in the “low” profile in Model 4. Again, the comparison of Model 2 and 4 was highly similar.

Of high interest, given the lack of overlap in measures uses to establish the profiles, is the comparison of Model 3 classifications (teacher-reported SR only) with Model 4 classifications (assessed EF). In this comparison, a total of 66% of children appeared to be classified similarly across the models – either in the low groups in both models or in the high group in Model 3 and the aligned moderate or high groups in Model 4. A further 11% of children were classified in the low profile by teacher SR report but in the moderate or high profiles by EF assessment. A further 22% of children were classified in the high profile by teacher report of SR, but in the low profile by EF assessment.

## Discussion

The aim of this study was to understand what profiles of SR and EF are discoverable in preschool children in low-income areas, using a range of indices for latent profile analyses involving both subcomponent and composite scores. Additionally, this study aimed to demonstrate how profile membership was related to other child outcomes including school readiness, visual motor integration, and social-emotional-behavioral development. Self-regulation was measured through three teacher-reported subscales (cognitive, behavioral, and emotional) and was either included in models as separate subscale scores (component measures) or collated together (composite measure). Executive function was assessed through assessments for inhibition, working memory, and shifting, which were also used as individual scores (component measure) or were collated together (composite measure). We ran four different latent profile models, with the first three models (including various component and composite scores for SR and EF) producing highly similar two-profile solutions with on average 36% of children in a low-skilled profile. The fourth model included only indices for the EF components and found a three-profile solution with a higher 52% of children in the low profile. The number of profiles found here was fewer than recent similar studies with preschool and kindergarten children where four or five-profile solutions were typical (Elliott, 2019; Litkowski et al., 2020; Bayly and Bierman, 2021). However, these studies generally had larger sample sizes, and in some cases included additional developmental measures beyond SR and EF (Elliott, 2019), or were not with low-income children (Litkowski et al., 2020). Kia-Keating et al. (2018) did produce a similar two-profile solution with 4- to 5-year-old children as the current study, using task assessments and parent report of SR, with 21% of children classified in a lower skilled profile.

### The Constellation of Self-Regulation and Executive Function Skills Within Profiles

The profile solutions presented here suggest that for this sample, SR and EF skills as measured by these indices at the beginning of the preschool year cluster together. That is, there were no solutions that identified a profile of children who showed, for example, strong EF, but poor SR, or vice versa. This differs somewhat to other recent profile analyses which have identified children with behavioral dysregulation without cognitive dysregulation (22%; Bayly and Bierman, 2021); children with average EF skills but poor academic and behavioral skills (23%; Elliott, 2019), and children with poor scores on direct assessments of EF but higher teacher-rated SR skills (7%; Litkowski et al., 2020). However, each of these studies used much larger sample sizes, different measures to the current study, and while Bayly and Bierman (2021) specifically sampled low-income children through Head Start programs in the United States, it is not clear to what extent the samples in any of these prior studies are similar in their level of social disadvantage and diversity compared to the current study. Further studies are needed with diverse samples and consistent measurement approaches to better understand the ways that SR and EF skills cluster together within individual children. Of note, when EF component scores were modeled (Model 4), the most distinguishing factor across profiles, which in turn were associated with school readiness and other outcomes, was children’s working memory scores (opposed to inhibition and shifting scores). This is aligned with prior work suggesting that of all the EFs, working memory is the most predictive of academic achievement (Ahmed et al., 2019). Of importance, is the fact that both in this study where profiles with relatively consistent skills across SR and EF measures were identified, and those prior that have found profiles with mixed skills, children with the poorest teacher-rated SR skills were at greatest risk of poor academic outcomes (Elliott, 2019; Litkowski et al., 2020; Bayly and Bierman, 2021). This has implications for the use of measures to identify children at most need of early support, which will be discussed further below.

### Prevalence of Self-Regulation and Executive Function Problems

Across the models, 31–52% of children were classified in the “low” performing profiles. These results compare similarly to other latent profile analysis studies which have investigated SR and EF in early childhood with on average approximately 30% of children classified into low profiles (Elliott, 2019; Litkowski et al., 2020; Bayly and Bierman, 2021). As participants from the current study were all children from socioeconomically disadvantaged community preschools, it is important to note that the profile label “high” used here is only relative to the performance of the sample. While 78% to 85% of children from the current sample scored close to the Australian average on teacher-reported measures of SR, a large portion of children underperformed for their age on the direct measures of EF. For example, 87% of children from this study scored below the 25^th^ percentile of Australian norms in working memory. Research from a larger and more representative population of children (*N*=2,880) estimated that 30% of Australian children fall within a “low” profile characterized by parent report of poorer attentional and emotional regulation that does not improve across birth to 5 years (Williams et al., 2016). Results from the current study with a low socioeconomic sample demonstrate a similar, if slightly higher estimate of children falling within a “low” SR profile (31%–52%). In this sample, SR and EF skills may have been impacted by the experience of socioeconomic disadvantage which can negatively impact children’s cognitive, social, and emotional development, physical health, language development, and pre-academic skills (Nicholson et al., 2010; Blair et al., 2015; Sharkins et al., 2017).

### Covariates Associated With Profile Membership.

Our findings also provide insight into the socio-demographic characteristics of the profiles found. Across all models, girls were more likely to be classified into the higher skilled profiles, in line with similar prior studies (Denham et al., 2012; Kia-Keating et al., 2018; Elliott, 2019). At the whole sample level here, bivariate correlations show that teachers rated girls as higher overall on teacher-reported behavioral and cognitive SR, but not emotional regulation, and that girls outperformed boys in shifting, but not in working memory or inhibition. While girls have demonstrated stronger EF and behavioral regulation skills (Veziroglu-Celik et al., 2018) as well as more prefrontal cortex activation during EF tasks (Shinohara and Moriguchi, 2020), other studies have shown no gender differences in assessed EF (Yamamoto and Imai-Matsumura, 2019). Indeed, it has been suggested that gender bias may influence the way teachers provide behavioral ratings of children’s SR, favoring girls (Yamamoto and Imai-Matsumura, 2019). Further studies are required to determine the degree to which teacher ratings do indeed show gender bias and to better understand profiles of SR and EF across genders.

The current study also found that children with developmental delay were more likely to be classified in the lower-performing profiles. This result reflects a prior study reporting that children with ADHD symptomatology were more likely to be classified into profiles characterized by EF deficits (Houwen et al., 2019). Further, children with non-English speaking home environments were more likely to be classified into the lower skilled profiles in the current study. It is possible that for some children, delivery of the EF tasks in English inhibited their performance. However, studies in the United States have found that low-income Spanish-speaking English language learners perform worse on EF assessments even when these are conducted in Spanish (McClelland and Wanless, 2012). Bivariate correlations here suggest that non-English speaking children performed worse than others on working memory and inhibition, but not on shifting. There was no evidence that teacher ratings of self-regulatory behavior differed by English language status. It is unclear in the context of this study where non-English speaking children spoke a wide range of languages at home, the extent to which the task format impacted on their performance, with further studies required.

### Profile Differences Across Motor, Social, and School Readiness Outcomes

This study also aimed to better understand how profile membership was linked with a range of important child outcomes. In line with prior research, results demonstrated that children classified in the higher performing profiles across all the models had on average significantly higher school readiness compared to children in the lower-performing profiles (Vitiello and Greenfield, 2017; Perry et al., 2018a; Bayly and Bierman, 2021). Additionally, children in higher performing profiles demonstrated significantly better visual motor integration skills, supporting understandings about the co-development of motor and cognitive skills, with children who lack adequate motor skills more likely to have problems in cognitive function (Cameron et al., 2012). Children in the current study who were classified in the higher performing profiles also demonstrated stronger teacher-reported prosocial and sociability behaviors and fewer internalizing and externalizing behaviors. While these results should be interpreted with caution given the shared method variance across teacher-report of SR, and these behavioral outcome measures, a similar gradient was also found across Model 4 where only assessed EF was used to profile children. Taken together, the findings are consistent with multiple prior studies that document the developmental importance of SR in terms of social skill and behavioral development (Williams and Berthelsen, 2017; Robson et al., 2020).

### Classification of Children Across Different Approaches to Profiling

The current study builds upon and extends previous literature by analyzing different models which incorporated either a composite or component measure of SR and EF. Models that used both SR and EF either as composite scores (Model 1), or component scores (Model 2), classified most children into the same profiles, with only 4.9% of children (*n*=10) differentially classified across these models. This finding provides additional support, within a person-centered context, for the increasing emphasis in the field on the consideration of EF as a unidimensional construct within early childhood (Hartung et al., 2020) and calls for the use of simple composite measures over latent variable and other measurement approaches (Willoughby and Blair, 2016; Camerota et al., 2020).

As there was a large amount of overlap in measures across models, the most interesting comparison is arguably across models where independent measures were used, specifically Model 3 (component measures of teacher-reported SR) and Model 4 (component measures of EF). However, it should be noted that as mentioned above, the modeling of individual components of EF is considered exploratory in nature here, given current best practice suggests a composite EF score is the most accurate representation of the construct in early childhood (Camerota et al., 2020). Across these models, a substantial percentage of the sample (66%) was classified in the same profiles (either low in both or higher performing in both). However, the remaining children in the sample were differentially classified across these two models (that is, low in one model but higher in the other). Although prior latent profile studies suggest that this constellation of mixed skills across SR and EF is discoverable in young children (Elliott, 2019; Litkowski et al., 2020; Bayly and Bierman, 2021), none of our models identified this group within the profiling process. Taken together, the findings suggest that for at least two-thirds of children here, groups of children identified as poor performing on teacher-report measures of SR are also performing poorly on measures of EF, school readiness, motor, and social outcomes. However, to be clear EF and SR are related but distinct constructs and the measures used in this study were distinct in their contexts. EF measures require performance on a cognitively demanding task while teacher measures reflected overall classroom behavior. Across models, profiles characterized by lower teacher report of SR had the poorest motor, school readiness, and social outcomes, concurring with and extending prior findings that teacher-report SR is a highly significant risk indicator for academic achievement, over and above measures of EF (Elliott, 2019; Litkowski et al., 2020; Bayly and Bierman, 2021).

### Implications

The findings of this study have several implications for policy and practice in the early years. Distinct profiles of SR and EF appear identifiable at the commencement of preschool, with poor skills across these areas not uncommon (over a third of this sample) and linked with risk for poorer skills in motor, school readiness, and social domains. This reinforces existing imperatives for a focus on SR in early childhood as a core target skill (Finders et al., 2021). Further, the performance of this sample of children in low socioeconomic areas compared to available norms reinforces existing and long-standing policy approaches that target low socioeconomic areas as priorities for early childhood support to reduce longer term risks (Solano and Weyer, 2017; National Head Start Association, 2021). A wide range of strategies including curriculum approaches (Diamond et al., 2019), early intervention programs (McClelland et al., 2019), and parenting programs (Magnuson and Schindler, 2019) can be used effectively to boost SR in young children and in doing so may provide a buffer against the negative effects of socioeconomic disadvantage (Crespo et al., 2019). More widespread uptake of evidence-based and ecologically appropriate approaches to building SR and EF skills is required, from as early as infancy, particularly in low socioeconomic communities.

In addition to universal approaches at the community level, it is imperative that children who may require additional support be identified as early as possible, with the findings of this study, and others, suggesting that educator-report of SR behaviors is an adequate approach. While it would be optimal to collect both direct child assessments and teacher-report data, it seems that teacher-report may be effective enough in gaining an overall sense of which children in the classroom have SR and EF challenges. Teacher-report data are time efficient and practical while direct cognitive assessments are not only costly and time-intensive but may offer limited incremental predictive validity in identifying developmental issues beyond behavioral ratings of SR (Tiego et al., 2019). Given that when EF scores were modeled here, working memory was the skill that differentiated children across low and high profiles, if a direct assessment is possible, it is recommended that working memory be considered a priority.

### Limitations and Future Directions

While this study makes an important and novel contribution though profiling of SR and EF using different indicators and approaches, in a low-income sample, it is not without its limitations. Our approach which involved profile classification followed by analysis relied on maximum-probability assignment of children into profiles, not accounting for uncertainty in latent profile membership. We ran a sensitivity analysis using the recently updated BCH approach in MPlus (Asparouhov and Muthén, 2021) and found: (1) number of profiles and assignment of children using profile probabilities did not change; (2) statistical significance of mean differences across our profiles on outcomes and covariates matched those reported in our analytic approach; and (3) estimated mean differences across profiles in relation to our outcome variables were more extreme than we report. This final point reflects the known pitfalls of our two-step classify-analyze approach, in which estimates of the mean differences across profiles tend to be somewhat attenuated, rather than over inflated (Lanza et al., 2013). Taken together, our results reported here are likely a conservative estimate of the group mean differences on socio-demographic and outcome variables across profiles. Future studies with larger sample sizes should endeavor to replicate these findings using the most sophisticated modeling approaches recommended at the time, which will allow for more nuanced understandings of the relations among the latent profile variable, predictors, and outcomes.

The cross-sectional design limits the extent to which changes over time and associations with more distal outcomes can be assessed. Future research could expand on this study by following children from a similar sample to better understand how SR and EF profiles develop over time, given it has been suggested that it is the *growth* in these critical SR skills which are essential for future outcomes (Howard and Williams, 2018). The study is also limited by the shared method variance across multiple teacher-report measures including SR indicators used in profiling, and the social outcome indices. However, associations among SR/EF profiles and assessed outcomes of motor skills and school readiness held as expected, increasing confidence in profile classifications. Finally, given that 87% of the sample performed below the 25th percentile for working memory, compared to population norms, it is possible that the EF measures selected here were not sensitive enough to capture adequate variation in performance in this group of children in low socioeconomic areas. Future studies should carefully consider selection of measures.

## Conclusion

This study documented profiles of self-regulation and executive function for a low-income sample at the beginning of their preschool year, and for the first time explored various approaches to profile specification through the use of different combinations of SR and EF measures. Findings suggest that SR and EF cluster together as a skillset in preschool children and that over 30% of children exhibit a profile of poorer skills with associated risk for poorer motor, school readiness, and social outcomes. Comparisons with available norms across all measures reinforce that children living in low socioeconomic areas are a critical target group for universal supports. Further, teacher report of SR appears a suitable approach to identify at-risk children with the poorest skills across both SR and EF. This approach is both time and cost-effective with the potential to identify which children would benefit from additional support to help combat the cyclic nature of socioeconomic disadvantage. Persistent efforts in this area of research and practice, for our most vulnerable children, are warranted if socioeconomic-based achievement and wellbeing gaps are to be adequately and economically addressed.

## Data Availability Statement

The raw data supporting the conclusions of this article will be made available by the authors, without undue reservation.

## Ethics Statement

The studies involving human participants were reviewed and approved by the Queensland University of Technology Human Research Ethics Committee. Written informed consent to participate in this study was provided by the participants’ legal guardian/next of kin.

## Author Contributions

KW conducted all analyses. KW and LB co-wrote the manuscript. All authors contributed to the article and approved the submitted version.

## Funding

KW is the recipient of an Australian Research Council Australian Discovery Early Career Award (project number DE190101096) funded by the Australian Government, which has supported this study.

## Conflict of Interest

The authors declare that the research was conducted in the absence of any commercial or financial relationships that could be construed as a potential conflict of interest.

## Publisher’s Note

All claims expressed in this article are solely those of the authors and do not necessarily represent those of their affiliated organizations, or those of the publisher, the editors and the reviewers. Any product that may be evaluated in this article, or claim that may be made by its manufacturer, is not guaranteed or endorsed by the publisher.
