# Supplementary material for: Latent Profiles of Teacher-Reported Self-Regulation and Assessed Executive Function in Low-Income Community Preschools: Relations With Motor, Social, and School Readiness Outcomes
Source: Front Psychol. 2021 Sep 27;12:708514. doi: 10.3389/fpsyg.2021.708514 (PMC8503553; doi:10.3389/fpsyg.2021.708514)
Supplement: Supplementary file 1 [file Table_1.docx]

Supplementary Table S1 Bivariate correlations and descriptive statistics for all variables used in analyses

|  |  | 1 | 2 | 3 | 4 | 5 | 6 | 7 | 8 | 9 | 10 | 11 | 12 | 13 | 14 | 15 | 16 | 17 | 18 | 19 | 20 | 21 |
| --- | --- | --- | --- | --- | --- | --- | --- | --- | --- | --- | --- | --- | --- | --- | --- | --- | --- | --- | --- | --- | --- | --- |
| 1 | Female | 1 |  |  |  |  |  |  |  |  |  |  |  |  |  |  |  |  |  |  |  |  |
| 2 | Child age | -.11 | 1 |  |  |  |  |  |  |  |  |  |  |  |  |  |  |  |  |  |  |  |
| 3 | Aboriginal | .03 | .01 | 1 |  |  |  |  |  |  |  |  |  |  |  |  |  |  |  |  |  |  |
| 4 | Non English home | -.14 | -.12 | -.20* | 1 |  |  |  |  |  |  |  |  |  |  |  |  |  |  |  |  |  |
| 5 | Development Delay | -.06 | .07 | -.02 | -.16* | 1 |  |  |  |  |  |  |  |  |  |  |  |  |  |  |  |  |
| 6 | Parent complete highschool | -.02 | -.16* | -.29 | .04 | -.10 | 1 |  |  |  |  |  |  |  |  |  |  |  |  |  |  |  |
| 7 | Parent low income | .13 | -.04 | .25* | .08 | -.12 | -.20* | 1 |  |  |  |  |  |  |  |  |  |  |  |  |  |  |
| 8 | EF | .14 | .29** | .00 | -.28** | -.10 | .09 | -.16 | 1 |  |  |  |  |  |  |  |  |  |  |  |  |  |
| 9 | WM | .09 | .23** | -.03 | -.22* | -.01 | .08 | -.09 | .73* | 1 |  |  |  |  |  |  |  |  |  |  |  |  |
| 10 | Inhibition | .05 | .25** | .13 | -.17* | -.17* | -.07 | -.08 | .78* | .41* | 1 |  |  |  |  |  |  |  |  |  |  |  |
| 11 | Shifting | .19* | .15* | -.11 | -.19 | .01 | .03 | -.19* | .68* | .12* | .30* | 1 |  |  |  |  |  |  |  |  |  |  |
| 12 | SR | .18* | .15* | .02 | -.10 | -.31* | .08 | -.11 | .43* | .34* | .32* | .21* | 1 |  |  |  |  |  |  |  |  |  |
| 13 | Beh SR | .17* | .15* | -.03 | -.13 | -.26* | .08 | -.15 | .49* | .43* | .36* | .32* | .90* | 1 |  |  |  |  |  |  |  |  |
| 14 | Cog SR | .18* | .09 | -.05 | -.08 | -.29* | .16* | -.15 | .38* | .28* | .28* | .27* | .89* | .74* | 1 |  |  |  |  |  |  |  |
| 15 | Emot SR | .12 | .15* | .12 | -.07 | -.29* | -.02 | -.01 | .26* | .20* | .21* | .19* | .87* | .68* | .63* | 1 |  |  |  |  |  |  |
| 16 | School ready | .00 | .20** | -.16* | -.16* | -.11 | .22* | -.17* | .56* | .45* | .47* | .30* | .38* | .41* | .34* | .25* | 1 |  |  |  |  |  |
| 17 | VMI | .09 | .29* | -.05 | -.04 | -.01 | -.01 | -.11 | .54* | .43* | .46* | .28* | .29* | .32* | .30* | .15* | .36* | 1 |  |  |  |  |
| 18 | Internalizing | -.13 | .02 | -.01 | .04 | .19* | -.00 | -.08 | -.17* | -.18* | -.09 | -.13 | -.55* | -.39* | -.53* | -.56* | -.07 | -.12 | 1 |  |  |  |
| 19 | Externalizing | -.11 | -.08 | -.06 | -.12 | .27* | .00 | .11 | -.35* | -.29* | -.29* | -.20* | -.80* | -.76* | -.55* | -.83* | -.28* | -.19* | .38* | 1 |  |  |
| 20 | Prosocial | .19* | .19* | -.04 | -.16* | -.29* | .03 | -.18* | .46* | .36* | .33* | .34* | .87* | .82* | .79* | .72* | .38* | .31* | -.53* | -.68* | 1 |  |
| 21 | Sociability | .15* | .17* | -.05 | -.20* | -.22* | .01 | -.03 | .32* | .17* | .21* | .34* | .54* | .39* | .57* | .47* | .19* | .24* | -.55* | -.30* | .71* | 1 |
|  | Range | .00-1.0 | 44-67 | 0.0-1.0 | 1.0-2.0 | .00-1.0 | .00-1.0 | .00-1.0 | -1.53-2.04 | -.91-2.20 | -2.07 - 2.10 | -3.19-2.65 | -2.65-.00 | -2.43-1.40 | -2.83-1.65 | -3.15-1.28 | 0-77 | 0-9 | 1 – 4.2 | 1 - 5 | 1 - 5 | 1 - 5 |
|  | Mean | .50 | 50.56 | .17 | 1.16 | .13 | .74 | .60 | .02 | .00 | .00 | .00 | .00 | .00 | .00 | .00 | 32.82 | 2.12 | 2.20 | 1.94 | 3.52 | 3.43 |
|  | SD | .50 | 4.47 | .38 | .37 | .34 | .44 | .49 | .73 | 1.00 | 1.00 | 1.00 | .89 | .10 | 1.00 | 1.00 | 16.73 | 1.87 | .66 | .93 | .95 | .90 |

EF = executive function composite; WM = working memory; SR = self-regulation composite; Beh SR = behavioral self-regulation; Cog SR = cognitive self-regulation; Emot SR = emotional self-regulation.
